# QTL analysis of tuber shape in a diploid potato population

**DOI:** 10.3389/fpls.2022.1046287

**Published:** 2022-11-10

**Authors:** Wei Huang, Jianke Dong, Xijuan Zhao, Zhiyuan Zhao, Chunyan Li, Jingcai Li, Botao Song

**Affiliations:** ^1^ Key Laboratory of Horticultural Plant Biology, Ministry of Education/Key Laboratory of Potato Biology and Biotechnology, Ministry of Agriculture and Rural Affairs/College of Horticulture and Forestry Sciences, Huazhong Agricultural University, Wuhan, Hubei, China; ^2^ Forestry and Fruit Research Institute, Wuhan Academy of Agricultural Sciences, Wuhan, Hubei, China; ^3^ College of Biology and Agricultural Resources, Huanggang Normal University/Hubei Key Laboratory of Economic Forest Germplasm Improvement and Resources Comprehensive Utilization, Huanggang, Hubei, China

**Keywords:** tuber shape, QTL-seq, self-pollinated, potato, marker development

## Abstract

Tuber shape is one of the most important traits for potato breeding. Since poor or irregular shape increases the difficulty of handling and processing, researching the inheritance of potato tuber shape for potato breeding is highly important. To efficiently identify QTL for tuber shape, a diploid potato population (PM7) was generated by self-pollinated M6 (*S. chacoense*). A QTL TScha6 for tuber shape was identified by the QTL-seq approach at 50.91-59.93 Mb on chromosome 6 in the potato DM reference genome. To confirm TScha6, four SSR and twenty CAPS markers around the QTL were developed and the TScha6 was narrowed down to an interval of ~ 1.85 Mb. The CAPS marker C6-58.27_665 linked to TScha6 was then used to screen 86 potato cultivars and advanced breeding lines. The tuber length/width (LW) ratio was significantly correlated with the presence/absence of C6-58.27_665, and the correlation coefficient was *r* = 0.55 (p < 0.01). These results showed that C6-58.27_665 could be applied in marker-assisted selection (MAS) for tuber shape breeding in the future. Our research sets the important stage for the future cloning of the tuber shape gene and utilities of the marker in the breeding program.

## Introduction

Potato (*Solanum tuberosum* L.) is the most important non-cereal food crop in the world, grown on 16.49 million hectares and consumed daily by more than one billion people (FAOSTAT, 2020). Tuber shape is one of the important morphological traits for the potato processing industry and fresh market use (Si et al., 2016; Stark et al., 2020). Poor and irregular shape increases the loss of peeling and leads to higher costs in the processing industry (Fan et al., 2022). Usually, the chipping industry tends to select round potato tubers, while French fries prefer long tubers (Bryan, 2011; Vreugdenhil et al., 2011). For consumers, the irregular tuber shape makes peeling difficult (Prashar et al., 2014). Thus, as the tuber shape influences the development of the processing industries and markets, breeding new potato varieties with uniform shapes is critical.

The variability in potato tuber appearance ranged from compressed to elongated, especially in the wild diploid potato (Lindqvist-Kreuze et al., 2015; Fan et al., 2022). Previous genetic studies have also shown that potato tuber shape was a quantitative trait in nature (De Jong and Burns, 1993). It has been long recognized that the tuber shape QTL (quantitative trait locus) included one major locus and some minor loci. The major shape QTL, termed *Ro*, was first reported by Van Eck et al. (1994) and identified on chromosome 10, explaining 75% of the observed variance for tuber shape. Similar mapping results were also reported in different potato germplasms in subsequent studies (ŚLiwka et al., 2008; Prashar et al., 2014; Endelman and Jansky, 2016; Fan et al., 2022; Pandey et al., 2022). The *Ro* gene was first mapped in the region 48.2-51.9 Mb on chromosome 10 of the potato DM reference genome (Endelman and Jansky, 2016), and the gene *StOFP20* was speculated to be the encoding gene of the *Ro* locus (Wu et al., 2018). In addition to the *Ro* locus, other genetic loci related to tuber shape have been reported. For example, Prashar et al. (2014) identified a major QTL for tuber shape on chromosome 2 by constructing a dense SNP (single nucleotide polymorphism) map and explaining 20% of the shape variation. Lindqvist-Kreuze et al. (2015) mapped a major QTL for tuber shape on chromosome 10, and two minor QTLs also associated with the trait on chromosomes 5 and 12, respectively. In cultivated Superior, tuber shape QTLs were reported on chromosomes 4, 6, 10, and 11 (Manrique-Carpintero et al., 2018).

Potato are polyploidy and self-incompatibility, which greatly limit the development of genetic studies in potato (Visser et al., 2009; Lindhout et al., 2011). It has long been recognized that cultivated potato is highly heterozygous autotetraploid (2n = 4x = 48) and the genetic ratios are complex because of reduced doubling in crosses (Zhou et al., 2014). Hence, most genetic analysis is conducted by using diploid potato populations (Li et al., 2005; Prashar et al., 2014; Torrance et al., 2020). Although progress was made at the diploid level, the F_1_ progeny segregated for potato traits due to two heterozygous diploid parents (Endelman and Jansky, 2016). It was difficult that produce an inbred line in the diploid potato population because of self-incompatibility (Pushkarnath, 1953; Pandey, 1962). However, Endelman and Jansky (2016) reported that a diploid inbred line F_2_ population was used for mapping tuber traits in potato. The F_2_ population was created by crossing between the double monoploid potato, DM1-3, and the S_7_ inbred line, M6 (Endelman and Jansky, 2016). This was the first report of the creation and application of an inbred line in potato, which indicates that the recombinant inbred line can also be developed in the future. Nonetheless, the genome sequence of M6 revealed that some loci were still heterozygous and two haplotypes are present (Jansky et al., 2014; Leisner et al., 2018). In addition, M6 till retained some excellent agronomic characteristics, including producing tubers, resistance PVA and PVY, and high dry matter content (Jansky et al., 2014; Huang et al., 2021).

In this study, a diploid potato population (PM7) was used to map potato tuber shape. The inbred parent of the population, designated M6, was inbred for seven generations (Jansky et al., 2014; Leisner et al., 2018). The PM7 population was constructed by self-crossing the M6. Then, we found that the population was segregated for tuber length/width (LW), and the QTL-seq approach was used to map the tuber shape in the population. The major QTL TScha6 for tuber shape was located on the distal long arm of potato chromosome 6 and delimited to a 1.85 Mb genomic region. Moreover, a CAPS marker linked to TScha6 was developed and evaluated for the capacity applied in marker-assisted selection (MAS) for tuber shape.

## Materials and methods

### Plant materials

The mapping population (PM7) was derived from self-pollinated diploid individual M6 (*S. chacoense*) which was inbred for seven generations and still retained some excellent agronomic characteristics (Jansky et al., 2014). The M6 genome revealed that some localized regions still maintained residual heterozygosity (Leisner et al., 2018).

The diploid self-cross population PM7 consists of a total of 180 individuals, 164 of which were used to construct the genetic map. The progeny was grown at Wuhan (30.5°N, 114.4°E) in three environments: spring 2016 (environment I), 2016 autumn (environment II), and 2017 spring (environment III). The field management ensured normal crop growth through standard agronomic practices and pesticide applications.

### Assessment of tuber shape

Tubers were harvested when most plants had become foliage senescence and phenotypic evaluation was carried out for tuber shape. All potato tubers were washed and five fully developed tubers per plot were selected for subsequent evaluation. Two methods were used for tuber shape assessment. On the one hand, tuber shape was assessed by visual evaluation from the total individuals on a 1-7 scale (Lindqvist-Kreuze et al., 2015). The 1-7 were represented as compressed (1), round (2), obovate (3), elliptic (4), oblong (5), long-oblong (6), and elongate (7). On the other hand, tuber shape was also evaluated by length/width (LW) ratio (Van Eck et al., 1994). For each PM7 progeny, the average of LW from five tubers was used as the trait value.

Additional 86 potato cultivars and advanced breeding lines constructed the natural population and were used to evaluate the effect of tuber shape QTL. These clones were also assessed the LW ratio as mentioned above. Generally, when the LW value is <1.4, the tuber shape is round; when the LW value is ≥1.4, the tuber shape is long (Ortiz and Huaman, 1994). The heritability of these traits were estimated according to Hara-Skrzypiec et al. (2018).

### QTL-seq analysis

Genomic DNA were isolated from frozen 500 mg of fresh leaves of the PM7 population using the cetyltrimethyl ammonium bromide (CTAB) method (Saghai-Maroof et al., 1985) and quantified using a Nanodrop 1000 spectrophotometer (Thermo Scientific).

For QTL-seq (Takagi et al., 2013), two pools, Round-pool and Long-pool were constructed by mixing an equal ratio of DNA. Each pool includes 24 individuals with extreme phenotype for tuber shape. The sequencing libraries were constructed using the NEBNext Ultra DNA Library Prep Kit (New England Biolabs, MA, USA) and subjected to 150 bp paired-end sequencing using an Illumina HiSeq 2500 platform in Tianjin Sequencing Center (Novegene Co., Ltd., Tianjin, China).

The quality of raw reads from Illumina HiSeq 2500 platform was first assessed by the FASTQC tool (http://www.bioinformatics.babraham.ac.uk/projects/fastqc/). Low-quality reads were discarded by the Trimmomatic tool (Lohse et al., 2012) with default settings and a Phred score cut-off of 20. High-quality clean reads were then aligned to the DM potato reference genome (Xu et al., 2011) using the BWA software with default settings (Li and Durbin, 2009). SAM files were converted to BAM files utilizing the SAMtools (Li et al., 2009). BAM files were then sorted and indexed by the Picard tool (http://broadinstitute.github.io/picard/). SNP calling was performed by the GATK software (McKenna et al., 2010; DePristo et al., 2011). Δ(SNP-index) and G’ were calculated by QTLseqr (Mansfeld and Grumet, 2018). The number of reads harboring SNPs that are different from the reference sequence is divided by the total reads as the SNP-index. The SNP-index of the Round-pool subtracted from the SNP-index of the Long-pool is Δ(SNP-index). The G statistic (G’) is calculated for each SNP based on the Nadaraya-Watson or tricube smoothing kernel (Nadaraya, 1964; Watson, 1964). The G’ and Δ(SNP-index) distribution were visualized by the sliding window method with 2 Mb window size and 10 kb increment (Takagi et al., 2013).

### Mapping QTL with SSR and CAPS markers

The simple sequence repeat (SSR) markers located in the candidate region (Xiao et al., 2018) were employed for polymorphism screening between the parental M6 and two pools (Round-pool and Long-pool). The polymorphic markers were then applied to all progeny. PCR mixtures (total 20 μl) containing 10 μl 2×Utaq PCR mix (Vazyme Co., Ltd., Nanjing, China), 1 μl of genomic DNA (50 ng/μl), 0.5 μl each forward and reverse primers (10 μmol/L), and 8 μl ddH_2_O were used for the following program: 3 min at 95 °C;35 cycles of 30 s at 95 °C, Tm °C at 30 s and 72 °C 30 s; finally, 10 min at 72 °C. The PCR products were checked on 6% polyacrylamide gel electrophoresis followed by silver staining (Xiao et al., 2018).

Based on filtered SNPs, the additional CAPS (cleaved amplified polymorphic sequences) markers were developed. Primers for the CAPS markers were designed with Snap Gene 4.1.6 program. SNPs were included within the restriction enzyme recognition site. The primers of CAPS markers were used for polymorphism screening between the parental M6 and two pools (Round-pool and Long-pool). PCR mixture and program were as same as SSR primers. After PCR amplification, PCR products were cleaved using restriction enzymes. The enzyme cleavage reaction system was as follows: 5 μl of PCR products; 0.05 U of restriction enzymes; 1μl of 10×reaction buffer; ddH_2_O for the rest. The enzyme cleavage mixtures were incubated at 37°C for 1 h. The reaction products were detected by agarose gel electrophoresis with a concentration of 0.8%. The size of the restriction fragment for each marker was visualized and recorded *via* the Bio-Rad Imaging System (Bio-Rad Laboratories). The linkage map was conducted with Joinmap^®^ 4 (Van Ooijen, 2006) and the parameter settings were referred to by Xiao et al. (2018). QTL analysis was conducted by MapQTL^®^6.0 based on the multiple-QTL models (MQM) (Van Ooijen, 2009). CAPS markers C6-57.1_554, C6-58.27_665, and C6_58.95_587 in MAS for tuber shape were dectected as mentioned above.

## Results

### Tuber shape analysis

**Figure 1:**
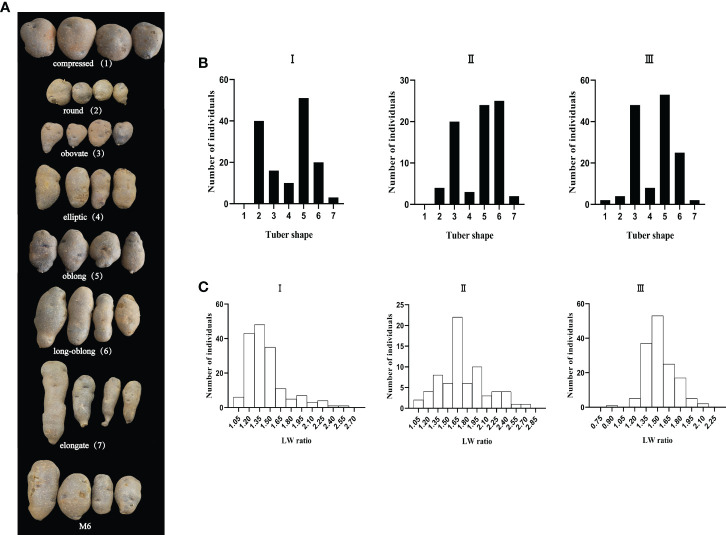
Phenotype of tuber shape in the PM7 population, and frequency distribution of tuber shape category and LW ratio in tree environment (environment I–III). **(A)** Photograph illustrating tuber shape variation in the PM7 population and the phenotype of the parent M6. Tuber shape was assessed on a 1-7 scale, where 1-7 were represented as compressed (1), round (2), obovate (3), elliptic (4), oblong (5), long-oblong (6), and elongate (7). **(B)** The frequency distribution of tuber shape category in the PM7 population in environment I–III. **(C)** The frequency distribution of LW ratio (the ratio of tuber length to width) in the PM7 population in environment I–III.

Tuber shape was assessed from the field-grown tubers of the PM7 population in 2016 and 2017 using the 1-7 scale and the LW method. The phenotypic data were provided in **Supplementary Table 1**. The mean LW of the parent M6 was 1.48, corresponding to elliptic tubers in all environments **(** **Figure 1A** **)**. In the population PM7, seven different shapes were included in three environments (**Figure 1A**), of which the most prevalent categories were round, obovate, oblong, and long-oblong (**Figure 1B**). The range of potato LW is 1.06-2.57 in environment I, 1.06-2.71 in environment II, and 0.89-2.11 in environment III (**Figure 1C**). The basic statistical data of the LW values in the PM7 population was shown in **Table 1**. The LW ratio shows a continuous distribution with skewness ranging from 0.53-1.49 and kurtosis from -0.11-2.26 in the three environments (**Table 1**). The LW values of environment I-III skewed positively and the tuber shape distributions in the three environments were concentrated to be round. The two assessing methods were significantly correlated (p < 0.01), and the Pearson correlation coefficients for the environment I-III were 0.588, 0.871, and 0.796, respectively (**Table 2**). The tuber shape in the PM7 population was demonstrated as a quantitative trait based on these phenotyping data in environment I-III. These phenotyping data were used for QTL analysis of tuber shape.

**Table 1:** Variance analysis of the LW values in three environments.

Trait	Environment	Mean	SD^a^	Variance	Heritability^b^	Skewness	Kurtosis
LW	I	1.44	0.29	0.08	0.69	1.49	2.26
	II	1.74	0.35	0.12	0.84	0.60	-0.11
	III	1.53	0.18	0.03	0.61	0.53	1.08

a the standard deviation in each environment.

b The broad-sense heritability estimated according to Hara-Skrzypiec et al. (2018).

**Table 2:** Correlation coefficients of the tuber shape traits in three environments.

Trait	Trait	LW			Tuber shape scale		
	Environment	I	II	III	I	II	III
LW	I	1	-0.05	0.08	.588^**^	0.03	0.07
	II		1	.238^**^	-0.08	.871^**^	.269^**^
	III			1	0.12	.287^**^	.796^**^
Tuber shape category	I				1	0.01	0.15
	II					1	.279^**^
	III						1

**p < 0.01.

### QTL-seq analysis for potato tuber shape

**Figure 2:**
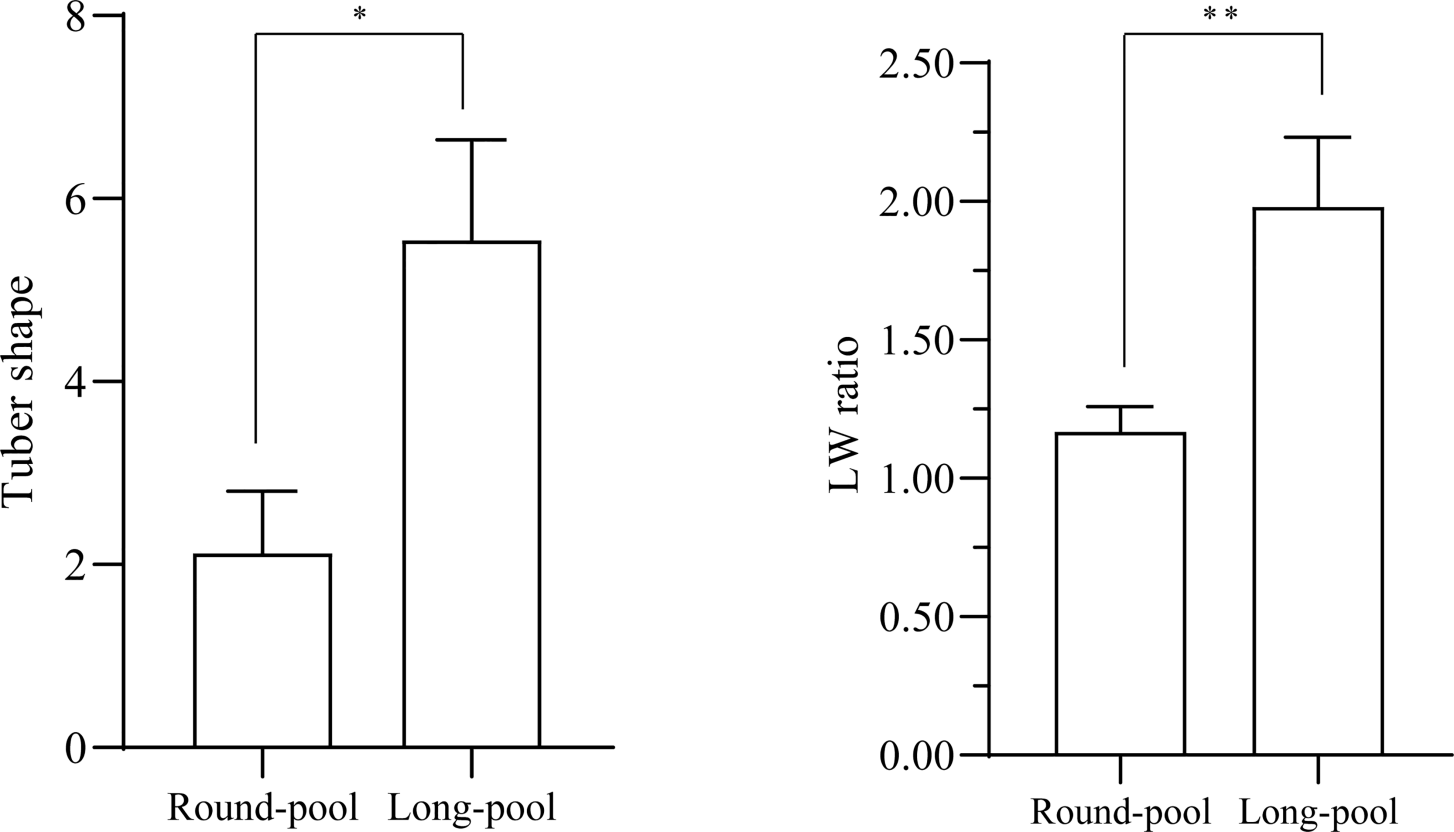
The difference analysis of tuber shape performance between Round-pool and Long-pool. **(A)** The mean value of the tuber shape category in the Round-pool was significantly lower than in the Long-pool (*p < 0.05 in the *t* test). **(B)** The mean value of the LW ratio in the Round-pool was very significantly lower than in the Long-pool (**p < 0.01 in Student *t* test).

Twenty-four individuals with the extreme long trait (LW ranged from 1.64-2.57) and 24 individuals with the extreme round trait (LW ranged from 1.06-1.23) were selected to prepare the Long-pool and Round-pool, respectively (**Figure 2**). After sequencing, 83,444,586 and 89,087,617 clean sequences were yielded from Round-pool and Long-pool, respectively. The mean value of Q20 and Q30 was 95.49% and 89.28%, respectively (**Supplementary Figure 1**). By BWA/SAM software, these sequences were aligned to the DM potato reference genome with an average alignment rate of 80.71%. The average genome coverage and depth were 95.56% and 32.67×, respectively.

**Figure 3:**
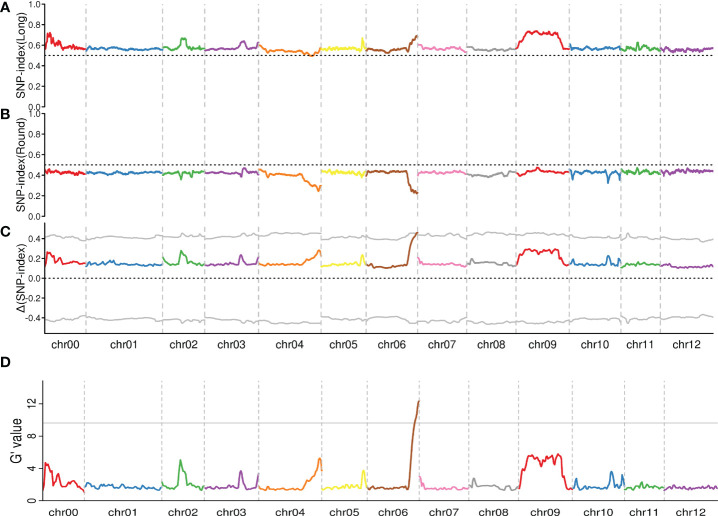
Identification of a genomic region corresponding to tuber shape by calculating the SNP-index and the G’ value. The x-axis represents the position of 12 chromosomes of the potato genome and the y-axis corresponds to the average SNP-index or G’ within a 2 Mb interval. **(A)** The SNP-index graph of the Long-pool. **(B)** The SNP-index of the Round-pool. **(C)** The Δ (SNP-index) graph, the grey dotted line represents that Δ (SNP-index) plot with 95% statistical two-sided confidence intervals under the null hypothesis of no QTL. **(D)** G’ graph, the grey line represents the p-values (0.01).

The two statistical methods of Δ(SNP-index) and G’ (Mansfeld and Grumet, 2018) were used to identify the QTL associated with tuber shape. According to the results of sequencing and reference sequence alignment, SNPs were detected by the GATK software and yielded 3,938,300 SNPs between the Long-pool and Round-pool. **Supplementary Figure 2** showed the distribution of all SNPs on the potato whole genome. The SNP-index of the Long-pool and Round-pool were calculated for each identified SNP and showed relationships with genomic positions by the sliding window method (**Figures 3A, B** **)**. We found that SNP-index graphs of the Long-pool and Round-pool showed contrasting patterns at the end of chromosome 6. Further, the Δ(SNP-index) was calculated by combining the information of the SNP-index from the Long-pool and Round-pool and showed the distribution on chromosomes 1-12 by the sliding window method (**Figure 3C**). The G’ of each identified SNP was calculated with the observed and expected allele depths and the value trends were plotted in **Figure 3D**. By combining the information of Δ(SNP-index) and the G’, we found that the chromosomal region of 50.91-59.53 Mb was associated with the tuber shape. Hence, the 8.62 Mb region of chromosome 6 distinguished by QTL-seq was considered as the candidate region for tuber shape, suggesting a tuber shape QTL across the 8.62 Mb region. The QTL was designated as TScha6 (tuber shape QTL on chromosome 6 of *S. chacoense* parent M6).

### Linkage mapping of TScha6

**Figure 4:**
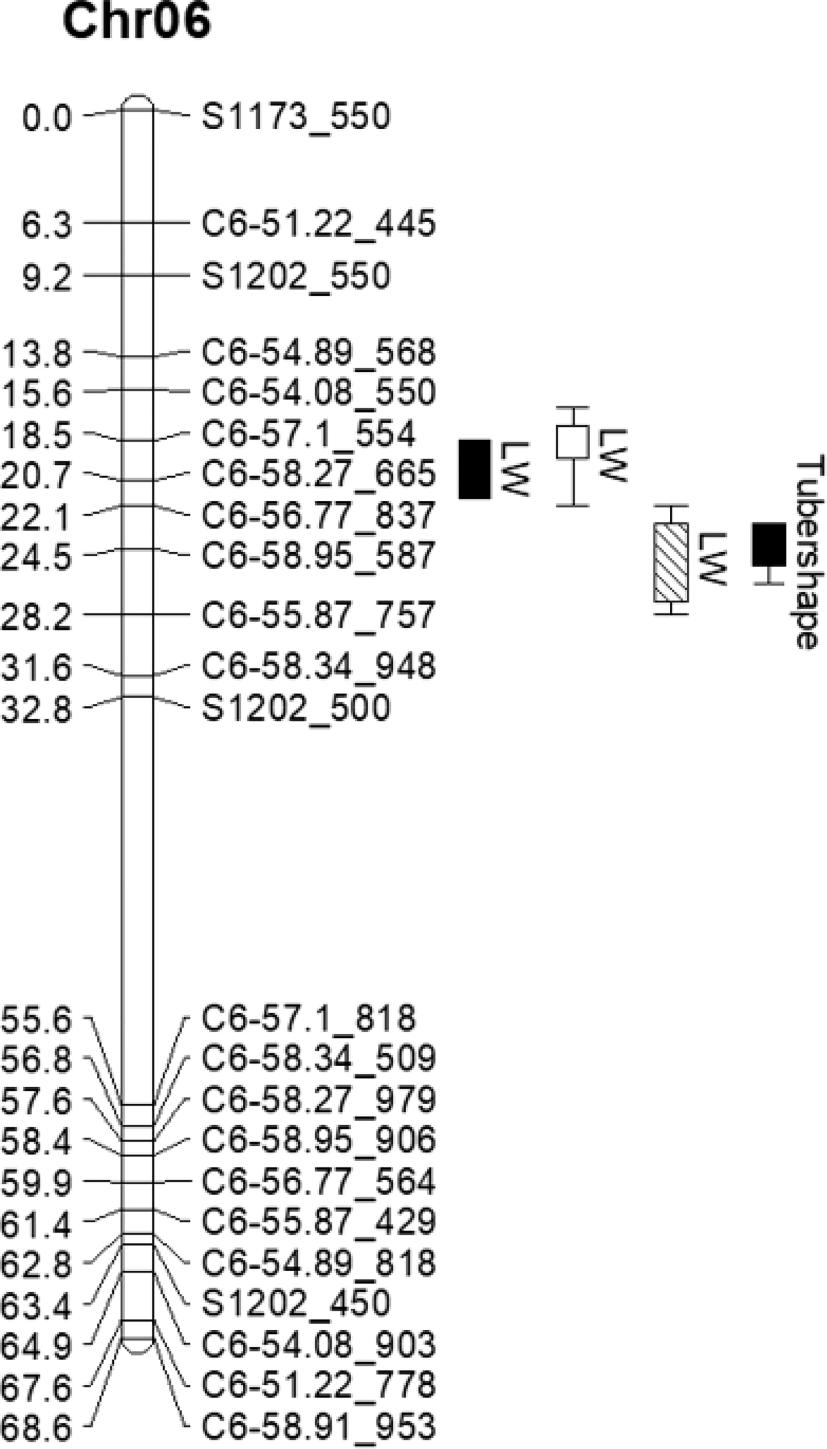
Identification and validation of the QTL Tscha6 for tuber shape in potato chromosome 6. On the linkage map, the maker locations are shown as cumulative distances in cM. The right side of the map shows the tuber shape QTL. Bars with different fill styles represent the tuber shape QTL in different environments. The solid bars represent environment I, the empty bar represents environment II, and the lower diagonal fill bar represents environment III).

To verify TScha6 detected by QTL-seq, a traditional method (linkage mapping) was applied to map the tuber shape in the PM7 population. The 11 sets of SSR primers (**Supplementary Table 2**) located around the candidate region were used to screen polymorphism between the Long-pool and Round-pool, and four polymorphic markers were obtained. To further define the interval on the linkage map of chromosome 6, SNPs were converted into CAPS markers. A total of 20 polymorphic CAPS markers (**Supplementary Table 3**) were obtained. These polymorphic markers were used to screen the population PM7, and a linkage map including four SSR markers and 19 CAPS markers was constructed. The total length of the map was 68.6 cM, with an average marker interval of 2.98 cM and a maximum distance between markers of 22.8 cM (**Figure 4**).

To narrow down the candidate region of tuber shape gene further, the traditional QTL analysis was performed based on the linkage map and the phenotypic data. QTL for the LW ratio was detected in all experiments and explained 10.2-17.5% of the phenotypic variation (**Table 3**). QTL for the tuber shape category was detected in only experiment I and explained 16.7% of the variation (**Table 3** and **Figure 4**). QTLs for the phenotypic variation were considered as the same QTL based on two-LOD support intervals. Hence, the tuber shape QTL was mapped between marker C6-57.1_554 and C6-58.95_587 (**Figure 4**), which was consistent with the 8.62 Mb region of TScha6 distinguished by QTL-seq and narrowed down the candidate region to a 1.85 Mb region.

**Table 3:** The QTL Tscha6 detecting for tuber shape by Multiple-QTL Models.

Trait	Environment	LOD	PVE (%)^a^	2-LOD interval (cM)	Peak (cM)	Peak Marker
LW	I	3.82	10.2	18.53-21.65	20.65	C6-58.27_665
	II	3.23	17.5	16.56-21.65	18.53	C6-57.1_554
	III	4.11	12.2	20.65-25.53	25.53	C6-58.95_587
Tuber shape category	I	5.54	16.7	23.11-26.53	24.53	C6-58.95_587

a The percentage of phenotypic variance explained by QTL.

### Tuber shape marker for MAS

**Figure 5:**
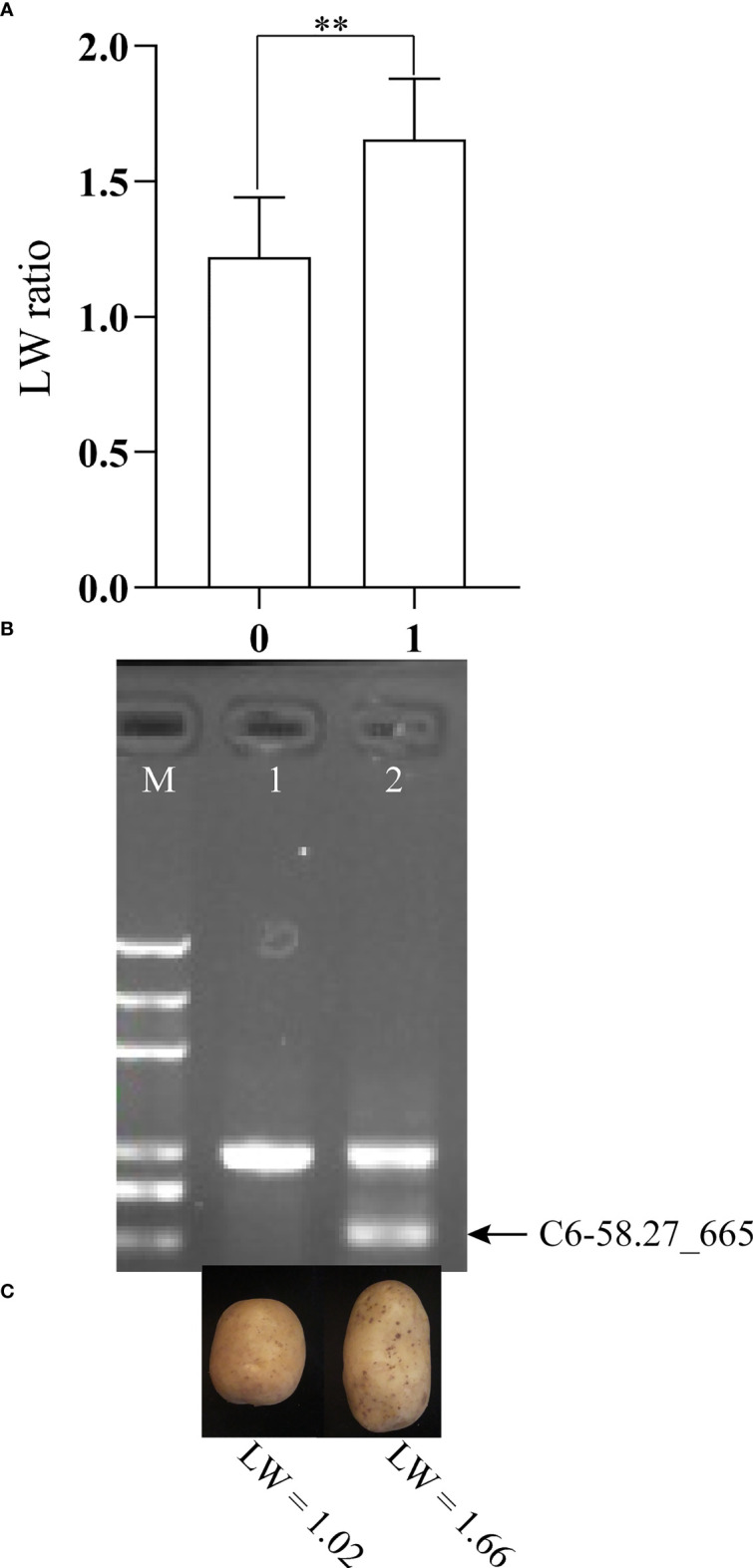
The effect of the tuber shape QTL was analyzed further by the occurrence of the QTL peak marker C6-58.27_665 in a natural population. **(A)** The difference analysis of LW value between 0 and 1. 0, the individuals lacked the marker C6-58.27_665; 1, the individuals carried the marker C6-58.27_665. **p < 0.01 in Student *t* test. **(B)** Detection of the CAPS marker C6-58.27_665 in natural polation. The PCR products were electrophoresed on 0.8% agarose gel. Lane M, 2 kb plus DNA ladder (Trans, China), and sizes of the ladders are 5000, 3000, 2000, 1000, 750, 500 bp, respectively. **(C)** The tuber shape of potato lines with (right) and without (left) C6-58.27_665.

A natural population, including 86 potato cultivars and advanced breeding lines, was used to verify TScha6. The LW ranged from 0.83 to 2.58 in the natural population. The tuber shape of seventeen clones with LW values (<1.4) was considered round, and the tuber shape of sixty-nine clones with high LW values (≥1.4) was considered long. According to the results of QTL mapping, CAPS markers C6-57.1_554, C6-58.27_665, and C6_58.95_587 were closely linked to the tuber shape gene. Therefore, the three markers were screened in natural populations. The correlation analysis showed that only the marker C6-58.27_665 was extremely significantly correlated with the LW in the natural population, and the correlation coefficient was 0.55 (p < 0.01). Further, the effect of the tuber shape QTL was analyzed by the occurrence of the QTL peak marker C6-58.27_665 in the natural population (**Figure 5**). The LW values of genotypes with the marker C6-58.27_665 were significantly higher than these of genotypes without the marker (p < 0.01). The coincidence of the phenotypic identification (round or long) and the marker C6-58.27_665 detection results reached 91.86% (**Table 4**). These results suggested that the marker C6-58.27_665 can be applied to molecular marker-assisted breeding for potato tuber shape.

**Table 4:** Evaluation of molecular markers C6-58.27_665 in the natural population.

Molecular marker C6-58.27_665^a^	Tuber shape	Clones	Total	Percentage (%)
0	Round (LW ≤ 1.4)	15	79	91.86
1	Long (LW > 1.4)	64		
0	Long (LW > 1.4)	5	7	8.14
1	Round (LW ≤ 1.4)	2		

a 0,The clones lacked the marker C6-58.27_665; 1, the clones carried the marker C6-58.27_665.

## Discussion

Potato is the most important non-cereal food crop in the world and is consumed daily by more than one billion people. Tuber shape is one of the major breeding objectives of breeders because poor and irregular shape increases the difficulty of handling and processing. Hence, it is necessary to research the inheritance of potato tuber shape. In this study, we employed the diploid population (PM7) derived from self-pollinated M6 with the elliptic tuber to conduct a genetic analysis. The progeny in the PM7 showed segregation in tuber shape. Using the strategy of traditional QTL mapping combined QTL-seq, we mapped a major QTL TScha6 for tuber shape in the population PM7 between 57.1 -58.95 Mb on chromosome 6 in the DM reference genome. Further, the effect of TScha6 was confirmed by a natural population.

QTL-seq (Takagi et al., 2013) could identify rapidly the candidate genomic region, which overcomes the difficulties of traditional methods in terms of labor, time and cost required. It has been successfully applied to mapping the QTL in cucumber, rice, and tomato (Takagi et al., 2013; Lu et al., 2014; Illa-Berenguer et al., 2015; Sun et al., 2018). In this study, we employed the method to identify a major QTL for tuber shape (**Figure 3**), which is consistent with the results of Manrique-Carpintero et al. (2018).

Tuber shape is quantitatively inherited and the trait was controlled by major or minor loci. The major QTL *Ro* on chromosome X was reported to control tuber shape (Chen et al., 2019). In addition, several minor QTLs have been identified, including QTLs on chromosome II (ŚLiwka et al., 2008; Prashar et al., 2014), chromosome V (Lindqvist-Kreuze et al., 2015), chromosome VI (Manrique-Carpintero et al., 2018) and chromosome XII (D’hoop et al., 2008). Manrique-Carpintero et al. (2018) identified four QTLs for tuber shape, one of which was also located at the end of chromosome 6 and the genetic distance between two flanking markers was 18 cM. In this study, TScha6 was mapped in a genetic interval of 6 cM, corresponding to a physical interval of ~ 1.85 Mb (**Figure 4**). We confirm that the candidate interval of TScha6 was smaller than that reported by Manrique-Carpintero et al. (2018). This is the first step towards the cloning of the tuber shape gene. In the future, the work will focus on fine mapping by expanding the population and developing additional markers.

Although most of the recent studies reported that the major genetic interval for tuber shape was mapped on chromosome 10, which hasn’t been detected by the QTL-seq in this study. This may be due to the following reasons. (1) The parent (M6) of the PM7 population is an S_7_ inbred clone but still has 0.26-2.10% heterozygous positions on the 12 chromosomes (Leisner et al., 2018). Thus, it was suggested that there may be a heterozygous segment related to tuber shape on the distal long arm of potato chromosome 6 in the M6 genome. (2) The tuber shape was controlled by the *Ro* locus on the chromosome X in the F_2_ population that was created by crossing between M6 × DM1-3 (Endelman and Jansky, 2016). In the F_2_ population, the female DM1-3 was long and the male M6 was elliptic while the F_1_ hybrid was oblong (Endelman and Jansky, 2016). The tuber shape trait in the F_2_ population was associated with the *StOFP20* marker (Wu et al., 2018). Hence, we speculated that the *Ro* locus in the M6 genome was homozygous and the locus for tuber shape on the chromosome couldn’t be detected in the PM7 population.

Marker-assisted selection (MAS) for the selection of plant breeding has been validated to be efficient in labor, time, and cost required (Xu and Crouch, 2008). This technology has been applied to select different traits in potato breeding, such as late blight resistance, PVY resistance, cold-induced sweetening, etc. (Nie et al., 2016; Chen et al., 2018; Xiao et al., 2018). However, although many QTLs of tuber shape was identified, there were few reports for tuber shape marker application. In this study, the CAPS marker C6-58.27_665 exhibited a high coincidence rate of 91.86% with the phenotypes of 86 potato breeding clones, indicating this marker is qualified for MAS of tuber shape.

## Conclusions

QTL for tuber shape was analyzed by the QTL-seq in diploid potato population PM7. A major QTL TScha6, explaining 10.2-17.5% of the variation of tuber shape, was identified on chromosome 6, and a CAPS marker linked to TScha6 was applied in marker-assisted selection (MAS) for tuber shape.

## Data availability statement

The data presented in the study are deposited in the BioProject database (ID PRJNA884166, http://www.ncbi.nlm.nih.gov/bioproject/884166).

## Author contributions

Conceived and designed the experiments: BS, JL, WH, JD. Performed the experiments: WH, JD, XZ, ZZ. Analyzed the data: WH, JD, CL. Prepared the manuscript: WH, BS, JL. Revised the manuscript: WH, JL, BS, JD. All authors contributed to the article and approved the submitted version.

## Funding

This project was funded by the Key Area Research and Development Program of Guangdong Province (2020B020219002) and the China Agriculture Research System of MOF and MARA (CARS-09-P07).

## Acknowledgments

The authors thank Ming Luo and Zhengnan Cheng for their technical support in the analysis of sequencing data.

## Conflict of interest

The authors declare that the research was conducted in the absence of any commercial or financial relationships that could be construed as a potential conflict of interest.

## Publisher’s note

All claims expressed in this article are solely those of the authors and do not necessarily represent those of their affiliated organizations, or those of the publisher, the editors and the reviewers. Any product that may be evaluated in this article, or claim that may be made by its manufacturer, is not guaranteed or endorsed by the publisher.

## References

[B1] Bryan G. J. (2011). “Mapping complex potato traits,” in The genetics, genomics, and breeding of potato. Eds. Bradeen J. M. Kole C. (New York, USA: CRC Press), 113–129.

[B2] Chen X. Lewandowska D. Armstrong M. R. Baker K. Lim T. Y. Bayer M.. (2018). Identification and rapid mapping of a gene conferring broad-spectrum late blight resistance in the diploid potato species *Solanum verrucosum* through DNA capture technologies. Theor. Appl. Genet. 131 (6), 1287–1297. doi: 10.1007/s00122-018-3078-6 29560514 PMC5945768

[B3] Chen N. Zhu W. Xu J. Duan S. Bian C. Hu J.. (2019). Molecular marker development and primary physical map construction for the tuber shape *Ro* gene locus in diploid potato (*Solanum tuberosum* l.). Mol. Breed. 39 (1), 6. doi: 10.1007/s11032-018-0913-z

[B4] De Jong H. Burns V. J. (1993). Inheritance of tuber shape in cultivated diploid potatoes. Am. Potato J. 70 (3), 267–284. doi: 10.1007/bf02849314

[B5] DePristo M. A. Banks E. Poplin R. Garimella K. V. Maguire J. R. Hartl C.. (2011). A framework for variation discovery and genotyping using next-generation DNA sequencing data. Nat. Genet. 43 (5), 491–498. doi: 10.1038/ng.806 21478889 PMC3083463

[B6] D’hoop B. B. Paulo M. J. Mank R. A. van Eck H. J. van Eeuwijk F. A. (2008). Association mapping of quality traits in potato (*Solanum tuberosum* l.). Euphytica 161 (1-2), 47–60. doi: 10.1007/s10681-007-9565-5

[B7] Endelman J. B. Jansky S. H. (2016). Genetic mapping with an inbred line-derived F_2_ population in potato. Theor. Appl. Genet. 129 (5), 935–943. doi: 10.1007/s00122-016-2673-7 26849236

[B8] Fan G. Wang Q. Xu J. Chen N. Zhu W. Duan S.. (2022). Fine mapping and candidate gene prediction of tuber shape controlling *Ro* locus based on integrating genetic and transcriptomic analyses in potato. Int. J. Mol. Sci. 23 (3), 1470. doi: 10.3390/ijms23031470 35163389 PMC8836246

[B9] FAOSTAT. (2020). Available at: https://crops.extension.iastate.edu/faostat.

[B10] Hara-Skrzypiec A. Śliwka J. Jakuczun H. Zimnoch-Guzowska E. (2018). QTL for tuber morphology traits in diploid potato. J. Appl. Genet. 59, 123–132. doi: 10.1007/s13353-018-0433-x 29492845 PMC5895667

[B11] Huang W. Nie B. Tu Z. Li C. Murphy A. M. Singh M.. (2021). Extreme resistance to potato virus a in potato cultivar Barbara is independently mediated by *Ra* and *Ry_sto_ * . Plant Dis. 105, 3344–3348. doi: 10.1094/PDIS-02-21-0233-SC 34096772

[B12] Illa-Berenguer E. Van Houten J. Huang Z. van der Knaap E. (2015). Rapid and reliable identification of tomato fruit weight and locule number loci by QTL-seq. Theor. Appl. Genet. 128 (7), 1329–1342. doi: 10.1007/s00122-015-2509-x 25893466

[B13] Jansky S. H. Chung Y. S. Kittipadukal P. (2014). M6: A diploid potato inbred line for use in breeding and genetics research. J. Plant Regist. 8 (2), 195–199. doi: 10.3198/jpr2013.05.0024crg

[B14] Leisner C. P. Hamilton J. P. Crisovan E. Manrique-Carpintero N. C. Marand A. P. Newton L.. (2018). Genome sequence of M6, a diploid inbred clone of the high-glycoalkaloid-producing tuber-bearing potato specie*s solanum chacoense*, reveals residual heterozygosity. Plant J. 94 (3), 562–570. doi: 10.1111/tpj.13857 29405524

[B15] Li X. Q. De Jong H. De Jong D. M. De Jong W. S. (2005). Inheritance and genetic mapping of tuber eye depth in cultivated diploid potatoes. Theor. Appl. Genet. 110 (6), 1068–1073. doi: 10.1007/s00122-005-1927-6 15719211

[B16] Li H. Durbin R. (2009). Fast and accurate short read alignment with burrows-wheeler transform. Bioinformatics 25 (14), 1754–1760. doi: 10.1093/bioinformatics/btp324 19451168 PMC2705234

[B17] Li H. Handsaker B. Wysoker A. Fennell T. Ruan J. Homer N.. (2009). The sequence alignment/map format and samtools. Bioinformatics 25 (16), 2078–2079. doi: 10.1093/bioinformatics/btp352 19505943 PMC2723002

[B18] Lindhout P. Meijer D. Schotte T. Hutten R. C. B. Visser R. G. F. van Eck H. J. (2011). Towards F_1_ hybrid seed potato breeding. Potato Res. 54 (4), 301–312. doi: 10.1007/s11540-011-9196-z

[B19] Lindqvist-Kreuze H. Khan A. Salas E. Meiyalaghan S. Thomson S. Gomez R.. (2015). Tuber shape and eye depth variation in a diploid family of Andean potatoes. BMC Genet. 16, 57. doi: 10.1186/s12863-015-0213-0 26024857 PMC4448561

[B20] Lohse M. Bolger A. M. Nagel A. Fernie A. R. Lunn J. E. Stitt M.. (2012). RobiNA: a user-friendly, integrated software solution for RNA-seq-based transcriptomics. Nucleic. Acids Res. 40 (Web Server issue), W622–W627. doi: 10.1093/nar/gks540 22684630 PMC3394330

[B21] Lu H. Lin T. Klein J. Wang S. Qi J. Zhou Q.. (2014). QTL-seq identifies an early flowering QTL located near *Flowering locus t* in cucumber. Theor. Appl. Genet. 127 (7), 1491–1499. doi: 10.1007/s00122-014-2313-z 24845123

[B22] Manrique-Carpintero N. C. Coombs J. J. Pham G. M. Laimbeer F. P. E. Braz G. T. Jiang J.. (2018). Genome reduction in tetraploid potato reveals genetic load, haplotype variation, and loci associated with agronomic traits. Front. Plant Sci. 9. doi: 10.3389/fpls.2018.00944 PMC6037889 30018631

[B23] Mansfeld B. N. Grumet R. (2018). QTLseqr: An r package for bulk segregant analysis with next-generation sequencing. Plant Genome 11 (2), 180006. doi: 10.3835/plantgenome2018.01.0006 PMC12810111 30025013

[B24] McKenna A. Hanna M. Banks E. Sivachenko A. Cibulskis K. Kernytsky A.. (2010). The genome analysis toolkit: A MapReduce framework for analyzing next-generation DNA sequencing data. Genome Res. 20, 1297–1303. doi: 10.1101/gr.107524.110 20644199 PMC2928508

[B25] Nadaraya E. A. (1964). On estimating regression. Theory. Probab. Appl. 10, 186–190. doi: 10.1137/1110024

[B26] Nie X. Sutherland D. Dickison V. Singh M. Murphy A. M. De Koeyer D. (2016). Development and validation of high-resolution melting markers derived from *Ry_sto_ * STS markers for high-throughput marker-assisted selection of potato carrying *Ry_sto_ * . Phytopathology 106 (11), 1366–1375. doi: 10.1094/PHYTO-05-16-0204-R 27442536

[B27] Ortiz R. Huaman Z. (1994). “Inheritance of morphological and tuber characteristics,” in Potato genetics. Eds. Bradshaw J. E. Mackay G. R. (Wallingford, United Kingdom: CAB International), 263–283.

[B28] Pandey K. K. (1962). Interspecific incompatibility in *Solanum* species. Am. J. Bot. 49, 874–882. doi: 10.1002/j.1537-2197.1962.tb15023.x

[B29] Pandey J. Vales M. I. Scheuring D. C. Koym J. W. (2022). Genomic regions associated with tuber traits in tetraploid potatoes and identification of superior clones for breeding purposes. Front. Plant Sci 13, 952263. doi: 10.3389/fpls.2022.952263 35937326 PMC9354404

[B30] Prashar A. Hornyik C. Young V. McLean K. Sharma S. K. Dale M. F.. (2014). Construction of a dense SNP map of a highly heterozygous diploid potato population and QTL analysis of tuber shape and eye depth. Theor. Appl. Genet. 127 (10), 2159–2171. doi: 10.1007/s00122-014-2369-9 25159608

[B31] Pushkarnath (1953). Studies on sterility in potato. Euphytica 2 (1), 49–58. doi: 10.1007/BF00035742

[B32] Saghai-Maroof M. A. Soliman K. M. Jorgensen R. A. Allard R. W. (1985). Ribosomal DNA spacer-length polymorphisms in barley: Mendelian inheritance, chromosomal location, and population dynamics. Proc Natl Acad Sci U.S.A. 81(24), 8014–8018. doi: 10.1073/pnas.81.24.8014 PMC392284 6096873

[B33] Si Y. Sankaran S. Knowles N. R. Pavek M. J. (2016). Potato tuber length-width ratio assessment using image analysis. Am. J. Potato Res. 94 (1), 88–93. doi: 10.1007/s12230-016-9545-1

[B34] ŚLiwka J. Wasilewicz-Flis I. Jakuczun H. Gebhardt C. (2008). Tagging quantitative trait loci for dormancy, tuber shape, regularity of tuber shape, eye depth and flesh colour in diploid potato originated from six *Solanum* species. Plant Breed. 127 (1), 49–55. doi: 10.1111/j.1439-0523.2008.01420.x

[B35] Stark J. C. Love S. L. Knowles N. R. (2020). “Tuber quality,” in Potato production systems. Eds. Stark J. C. Thornton M. Nolte P. (United States: Springer International Publishing), 479–497.

[B36] Sun J. Yang L. Wang J. Liu H. Zheng H. Xie D.. (2018). Identification of a cold-tolerant locus in rice (*Oryza sativa* l.) using bulked segregant analysis with a next-generation sequencing strategy. Rice (N Y) 11 (1), 24. doi: 10.1186/s12284-018-0218-1 29671148 PMC5906412

[B37] Takagi H. Abe A. Yoshida K. Kosugi S. Natsume S. Mitsuoka C.. (2013). QTL-seq: rapid mapping of quantitative trait loci in rice by whole genome resequencing of DNA from two bulked populations. Plant J. 74 (1), 174–183. doi: 10.1111/tpj.12105 23289725

[B38] Torrance L. Cowan G. H. McLean K. MacFarlane S. Al-Abedy A. N. Armstrong M.. (2020). Natural resistance to potato virus y in *Solanum tuberosum* group phureja. Theor. Appl. Genet. 133 (3), 967–980. doi: 10.1007/s00122-019-03521-y 31950199 PMC7021755

[B39] Van Eck H. J. Jacobs J. M. Stam P. Ton J. Stiekema W. J. Jacobsen E. (1994). Multiple alleles for tuber shape in diploid potato detected by qualitative and quantitative genetic analysis using RFLPs. Genetics 137 (1), 303–309. doi: 10.1093/genetics/137.1.303 7914504 PMC1205946

[B40] Van Ooijen J. W. (2006). JoinMap 4, software for the calculation of genetic linkage maps in experimental populations (Wageningen: Kyazma B V).

[B41] Van Ooijen J. W. (2009). MapQTL6, software for the mapping of quantitative trait in experiment populations of diploid species (Wageningen: Kyazma B V).

[B42] Visser R. G. F. Bachem C. W. B. de Boer J. M. Bryan G. J. Chakrabati S. K. Feingold S.. (2009). Sequencing the potato genome: Outline and first results to come from the elucidation of the sequence of the world’s third most important food crop. Am. J. Potato Res. 86 (6), 417–429. doi: 10.1007/s12230-009-9097-8

[B43] Vreugdenhil D. Bradshaw J. Gebhardt C. Govers F. Taylor M. A. MacKerron D. K.. (2011). Potato biology and biotechnology: advances and perspectives (Amsterdam: Elsevier Press).

[B44] Watson G. (1964). Smooth regression analysis. Sankhy=a: Indian J. Statistics Ser. A 26, 359–372. Available at: https://pub.uni-bielefeld.de/record/2715511

[B45] Wu S. Zhang B. Keyhaninejad N. Rodriguez G. R. Kim H. J. Chakrabarti M.. (2018). A common genetic mechanism underlies morphological diversity in fruits and other plant organs. Nat. Commun. 9 (1), 4734. doi: 10.1038/s41467-018-07216-8 30413711 PMC6226536

[B46] Xiao G. Huang W. Cao H. Tu W. Wang H. Zheng X.. (2018). Genetic loci conferring reducing sugar accumulation and conversion of cold-stored potato tubers revealed by QTL analysis in a diploid population. Front. Plant Sci. 9. doi: 10.3389/fpls.2018.00315 PMC5854652 29593769

[B47] Xu Y. Crouch J. H. (2008). Marker-assisted selection in plant breeding: From publications to practice. Crop Sci. 48 (2), 391–407. doi: 10.2135/cropsci2007.04.0191

[B48] Xu X. Pan S. Cheng S. Zhang B. Mu D. Ni P.. (2011). Genome sequence and analysis of the tuber crop potato. Nature 475 (7355), 189–195. doi: 10.1038/nature10158 21743474

[B49] Zhou J. Fang H. Shan J. Gao X. Chen L. Xie C.. (2014). A major QTL located on chromosome V associates with *in vitro* tuberization in a tetraploid potato population. Mol. Genet. Genomics 289 (4), 575–587. doi: 10.1007/s00438-014-0832-6 24619101

