# Supplementary figures and images for: QTL analysis of tuber shape in a diploid potato population
Source: Front Plant Sci. 2022 Nov 10;13:1046287. doi: 10.3389/fpls.2022.1046287 (PMC9685338; doi:10.3389/fpls.2022.1046287)

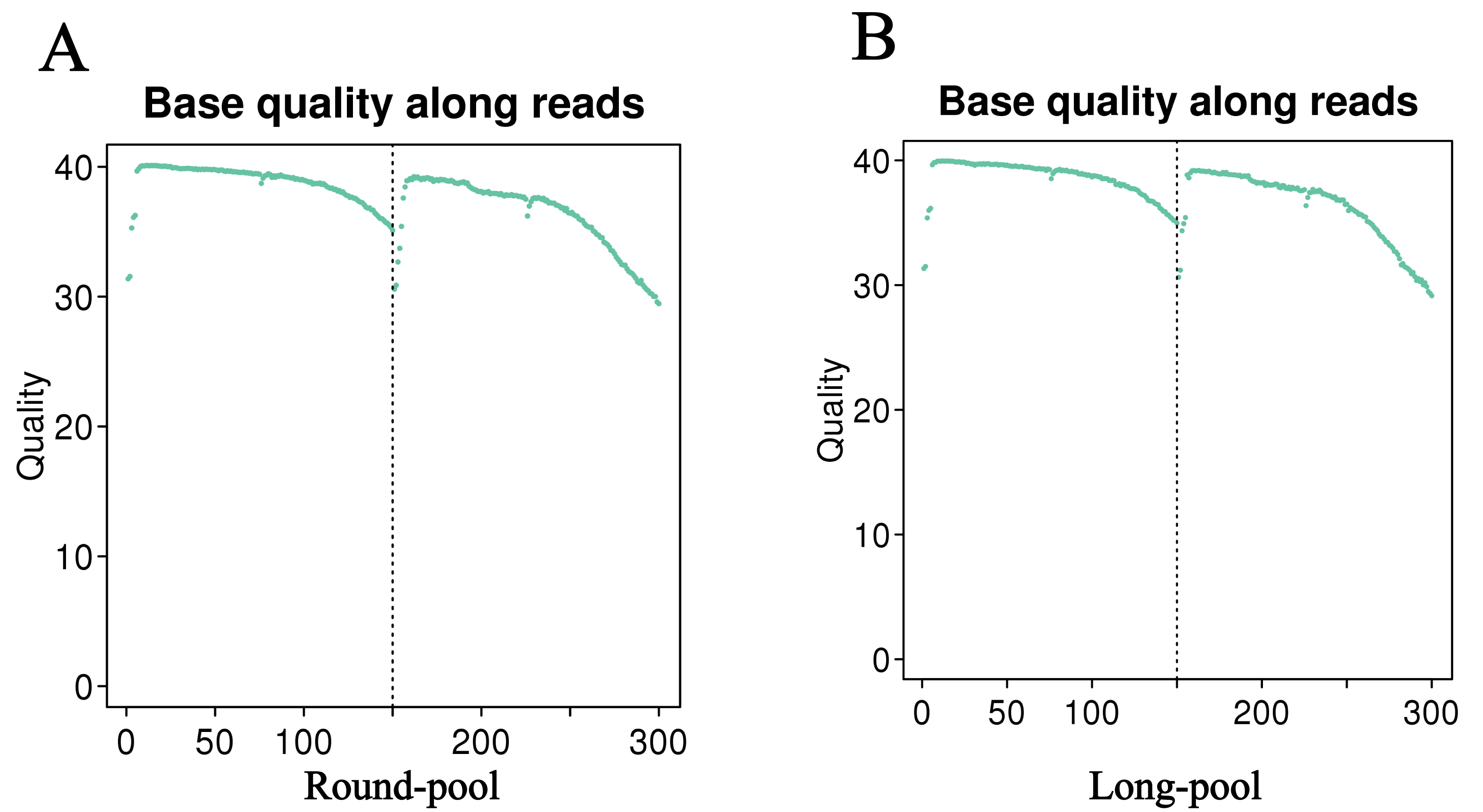

Supplement: Supplementary file 1 [file Image_1.tif]

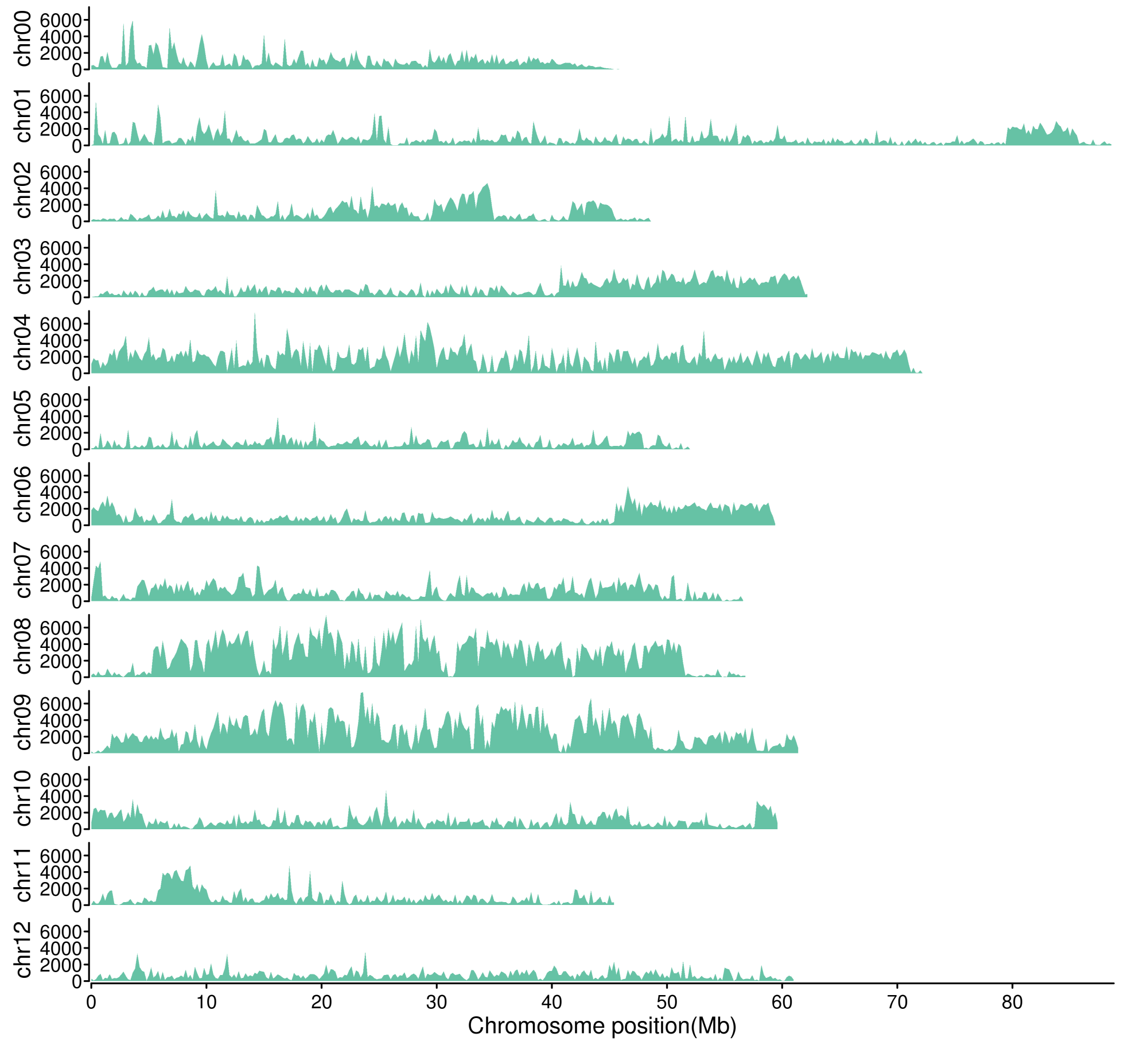

Supplement: Supplementary file 2 [file Image_2.tif]
